# Preoperative Serum Fibrinogen is Associated With Acute Kidney Injury after Cardiac Valve Replacement Surgery

**DOI:** 10.1038/s41598-020-63522-6

**Published:** 2020-04-14

**Authors:** Jing Juan Yang, Wen Hua Lei, Peng Hu, Bin Bin Wu, Jian Xiao Chen, Yi Ming Ni, En Yin Lai, Fei Han, Jiang Hua Chen, Yi Yang

**Affiliations:** 1 Department of Nephrology, the Fourth Affiliated Hospital, Zhejiang University School of Medicine, N1 Shangcheng Road, Yiwu, China; 2 Kidney Disease Center, the First Affiliated Hospital, Zhejiang University School of Medicine; Key Laboratory of Kidney Disease Prevention and Control Technology, Zhejiang Province; The Third Grade Laboratory under the National State, Administration of Traditional Chinese Medicine, 79 Qingchun Road, Hangzhou, China; 3 Department of Cardiothoracic Surgery, the First Affiliated Hospital, Zhejiang University School of Medicine, 79 Qingchun Road, Hangzhou, China; 4 Department of Physiology, School of Basic Medical Sciences, Zhejiang University School of Medicine, 866 Yuhangtang Road, Hangzhou, China

**Keywords:** Biomarkers, Cardiology, Nephrology

## Abstract

Acute kidney injury (AKI) after open cardiac surgery is associated with a longer hospital stay and higher risk of mortality. We aimed to explore the association between preoperative serum fibrinogen level and risk of postoperative AKI in patients with open cardiac surgery. 3459 patients who underwent cardiac valve replacement surgery from January 2011 to September 2015 were recruited. The primary outcome was AKI, defined as AKI stage-1 or higher based on the Kidney Disease: Improving Global Outcomes (KDIGO) Guidelines. Synthetic Minority Oversampling Technique (SMOTE) was used to subsample minority groups to eliminate classification bias. 510 (14.74%) patients developed postoperative AKI. Serum fibrinogen was independently associated with AKI (OR = 1.211, 95% CI 1.080 to 1.358, p = 0.001) after adjustment of covariates. The receiver operator characteristic (ROC) curve for the outcome of AKI, after the addition of serum fibrinogen, had a c-statistic increasing from 0.72 to 0.73 (p < 0.001). This translated to a substantially improved AKI risk classification with a net reclassification index of 0.178 (p < 0.001). After SMOTE subsampling, serum fibrinogen was still independently associated with AKI grade 1 or higher (OR = 1.212, 95% CI 1.1089 to 1.347, p = 0.003). Preoperative serum fibrinogen levels were associated with the risk of postoperative AKI after cardiac valve replacement surgery.

## Introduction

Acute kidney injury (AKI) is an adverse postoperative complication of cardiac valve surgery and occurs in 3–30% of patients after surgery, which is independently associated with a longer hospital stay and higher risk of short- and long-term mortality^1,2^. The pathophysiology of AKI is complex and multifactorial, including nephrotoxins, hypoxia, mechanical trauma, inflammation, cardiopulmonary bypass (CPB) and hemodynamic instability^3^. However, there are still no well-defined, effective pharmacological strategies for both prevention and treatment of AKI in the setting of cardiac valve replacement surgery. Recognizing and alleviating risk factors preoperatively and optimizing intraoperative practices may, to a great extent, reduce the incidence of AKI. A full understanding and scrutinizing of the preoperative AKI risk provides an opportunity for clinicians to optimize high risk patients and to initiate preventative and therapies.

Cardiac valve replacement surgery has its unique features comparing with non-cardiac surgery, including CPB, aorta cross-clamping, and high rates and volumes of exogenous blood product transfusion, which may increase the risk of AKI^4^. Several risk tools have been developed to predict postoperative AKI after cardiac surgery, such as the Cleveland Clinic score^5^, the Metha score^6^, the Simplified Renal Index score^7^ and the Birnie score^8^. These scores have limited general clinical application due to several weaknesses, such as non-consensus AKI definitions and different races. Therefore, more efficient and sensitive risk predictors are imperative to identify patients with high risk of AKI.

Fibrinogen is a soluble glycoprotein and is an important regulator of coagulation, inflammation, wound healing and angiogenesis *via* interactions with blood cells, endothelial cells and other cell types^9–11^. For managing postoperative bleeding, fibrinogen concentrate is conventionally used as the standard replacement in many European countries with the exception of United Kingdom^12^. Previous study has demonstrated that the prophylactic infusion of fibrinogen concentrate reduces the bleeding after coronary artery bypass surgery and other types of cardiac surgery^13^. However, a randomized study found that prophylactic fibrinogen increased the incidence of AKI after heart transplantation^14^.

Fibrinogen is recognized as an acute-phase response protein, and it increases several-fold during inflammation or tissue damage^15^. Several studies have found that serum or urinary fibrinogen is associated with contrast-induced nephropathy^16^ and sepsis-associated AKI^17^.

Therefore, it is necessary to further investigate the association between fibrinogen concentrate and adverse outcomes of patients undergoing cardiac surgery, especially the association between preoperative baseline serum fibrinogen and postoperative AKI. Thus, the purpose of the present study was to explore the association between the preoperative serum fibrinogen level and risk of postoperative AKI in patients with cardiac valve replacement surgery.

## Results

### Baseline characteristics

The mean age (±standard deviation) of patients in the study cohort was 54.18 ± 11.34 years, and 1525 patients (44.09%) were male. Among the operation procedures, 95.63% (n = 3308) were valve replacement surgery only, and 4.37% (n = 151) were combined valve replacement surgery and others. A total of 1.19% of the surgeries were emergency and 2.89% were reoperations. We compared baseline characteristics between patients with and without postoperative AKI. The most significant differences were that patients with postoperative AKI were older, male, more likely to have hypertension, diabetes mellitus, cerebrovascular disease and obesity, more likely to have poor left ventricular ejection fraction (LVEF), lower estimated glomerular filtration (eGFR) and anemia, and more likely to undergo reoperations and a combined valve replacement surgery and others. Patients with postoperative AKI had a longer cardiopulmonary bypass and aortic cross-clamp time, higher mortality and longer in-hospital stay (Table 1). Serum fibrinogen was higher in patients with postoperative AKI subsequent to cardiac valve replacement surgery (2.95 ± 0.91 vs. 2.72 ± 0.79; p = 0.002).

**Table 1:** Baseline characteristics of patients undergoing Cardiac Valve Replacement Surgery.

Variables	Overall (n = 3459)	AKI (n = 510)	Non-AKI (n = 2949)	P Value
**Age (years)**				<0.001
≤60	2370 (68.52)	271 (53.14)	2099 (71.18)	
61–75	1048 (30.30)	224 (43.92)	824 (27.94)	
>75	41 (1.19)	15 (2.94)	26 (0.88)	
**Male (%)**	1525 (44.09)	291 (57.06)	1234 (41.84)	<0.001
**Co-morbidities**				
Hypertension (%)	786 (22.72)	183 (35.88)	603 (20.45)	<0.001
Diabetes Mellitus (%)	169 (4.89)	48 (9.41)	121 (4.10)	<0.001
Cerebrovascular Disease (%)	210 (6.07)	43 (8.43)	167 (5.66)	0.016
Obesity (%)	287 (8.30)	69 (13.53)	218 (7.39)	<0.001
**Surgical Characteristics**				
Prior Cardiac Surgery (%)	100 (2.89)	34 (6.67)	66 (2.24)	<0.001
Emergent Surgery (%)	41 (1.19)	9 (1.76)	32 (1.09)	0.190
**Surgery type**				<0.001
Valve replacement only(%)	3308 (95.63)	455 (89.22)	2853 (96.74)	
Combined Valve replacement and others (%)	151 (4.37)	55 (10.78)	96 (3.26)	
**Aortic cross-clamp time (min)**	53.19 ± 25.74	60.67 ± 29.75	51.94 ± 24.79	<0.001
**Cardiopulmonary bypass time (min)**	65.73 ± 30.35	77.03 ± 41.94	63.84 ± 27.51	<0.001
**Preoperative LVEF**				0.003
Good (>50.00%)	3036 (87.77)	444 (87.06)	2592 (87.89)	
Fair (30.01–50.00%)	421 (12.17)	64 (12.55)	357 (12.11)	
Poor (≤30.00%)	2 (0.06)	2 (0.39)	0 (0.00)	
**Preoperative serum creatinine (umol/L)**	73.10 ± 33.20	88.74 ± 73.17	70.40 ± 17.86	<0.001
**Preoperative eGFR (mL/min/1.73 m**^**2**^**)**				<0.001
≤30.00	14 (0.40)	10 (1.96)	4 (0.14)	
30.01–60.00	194 (5.61)	78 (15.29)	116 (3.93)	
60.01–90.00	1181 (34.14)	214 (41.96)	967 (32.79)	
>90.00	2070 (59.84)	208 (40.78)	1862 (63.14)	
**Preoperative HB (g/L)**				0.001
≤100	196 (5.67)	42 (8.24)	154 (5.22)	
101–120	628 (18.16)	109 (21.37)	519 (17.60)	
>120	2635 (76.18)	359 (70.39)	2276 (77.18)	
**Preoperative INR**				0.009
≤0.90	167 (4.83)	21 (4.12)	146 (4.95)	
0.91–1.10	2294 (66.32)	313 (61.37)	1981 (67.18)	
>1.10	998 (28.85)	176 (34.51)	822 (27.87)	
**Preoperative fibrinogen (g/L)**	2.75 ± 0.81	2.95 ± 0.91	2.72 ± 0.79	0.002
**Low level of fibrinogen (<1.8 g/L) (%)**	182 (5.26)	28 (5.49)	154 (5.22)	
**Normal level of fibrinogen (1.8–3.5 g/L) (%)**	2818 (81.47)	384 (75.29)	2434 (82.54)	
**Elevated level of fibrinogen (>3.5 g/L) (%)**	459 (13.27)	98 (19.22)	361 (12.24)	
**Length of hospital stay (days)**	16.90 ± 7.88	20.67 ± 12.53	16.25 ± 6.54	<0.001
**Overall Mortality (%)**	31 (0.90)	23 (4.51)	8 (0.27)	<0.001

Note: continuous variables shown as mean ± SD; categorical variables as number (percentage).

Abbreviations: acute kidney injury (AKI); left ventricular ejection fraction (LVEF); estimated glomerular filtration (eGFR); hemoglobin (HB); international normalized ratio (INR).

### Association of serum fibrinogen with postoperative AKI

Among all participants, 510 (14.74%) had AKI and 90 (2.60%) had severe AKI. Most preoperative risk factors were independently associated with AKI (Table 2). After adjustment for covariates, serum fibrinogen was independently associated with AKI grade 1 or higher (OR = 1.211, 95% CI 1.100 to 1.333, p = 0.001), that is, every 1 g/L increase in serum fibrinogen level increased the AKI risk by 21.1%. There was no association between serum fibrinogen and severe AKI defined as grade 2 or higher (OR = 1.193, 95% CI 0.828 to 1.719, p = 0.343) (Table 3).

**Table 2:** Preoperative risk factors and AKI Risk Following Cardiac Valve Replacement Surgery.

	Without SMOTE			With SMOTE		
	Odds Ratio	95% CI	P Value	Odds Ratio	95% CI	P Value
**Age (years)**						
≤60	1.000			1.000		
61–75	1.442	1.193–1.742	0.001	1.413	1.147–1.737	0.006
>75	2.068	1.126–3.704	0.044	1.284	0.590–2.621	0.579
**Male (%)**	1.949	1.635–2.327	<0.001	1.795	1.480–2.180	<0.001
**Co-morbidities**						
Hypertension (%)	1.571	1.287–1.914	<0.001	1.666	1.340–2.067	<0.001
Diabetes Mellitus (%)	1.563	1.083–2.234	0.042	1.476	0.981–2.192	0.111
Cerebrovascular Disease (%)	1.447	1.046–1.975	0.056	1.216	0.839–1.729	0.372
Obesity (%)	1.024	0.744–1.396	0.900	0.993	0.699–1.396	0.974
**Surgical Characteristics**						
Prior Cardiac Surgery (%)	3.576	2.400–5.270	<0.001	3.786	2.411–5.865	<0.001
Emergent Surgery (%)	0.818	0.370–1.683	0.662	0.883	0.367–1.943	0.804
**Surgery type**						
Valve replacement only (%)	0.423	0.306–0.588	<0.001	0.402	0.282–0.577	<0.001
**Preoperative LVEF**						
Good (>50.00%)	1.000			1.000		
Fair (30.01–50.00%)	0.000	0.000	0.948	0.000	0.000	0.965
Poor (≤30.00%)	0.000	0.000	0.947	0.000	0.000	0.965
**Preoperative eGFR (mL/min/1.73 m**^**2**^**)**						
≤30.00	1.000			1.000		
30.01–60.00	0.255	0.083–0.692	0.031	0.286	0.090–0.827	0.059
60.01–90.00	0.095	0.031–0.251	<0.001	0.126	0.041–0.354	0.001
>90.00	0.058	0.019–0.153	<0.001	0.763	0.025–0.214	<0.001
**Preoperative HB (g/L)**						
≤100	1.000			1.000		
101–120	0.804	0.557–1.173	0.335	0.733	0.488–1.114	0.215
>120	0.572	0.409–0.810	0.007	0.567	0.392–0.834	0.013
**Preoperative INR**						
≤0.90	1.000			1.000		
0.91–1.10	1.040	0.697–1.605	0.875	0.965	0.625–1.547	0.897
>1.10	1.291	0.848–2.024	0.334	0.076	0.739–1.905	0.590
**Preoperative fibrinogen (g/L)**	1.211	1.100–1.333	0.001	1.212	1.089–1.347	0.003

Abbreviations: synthetic minority oversampling technique (SMOTE); acute kidney injury (AKI); coronary artery bypass graft (CABG); left ventricular ejection fraction (LVEF); estimated glomerular filtration (eGFR); hemoglobin (HB); international normalized ratio (INR).

**Table 3:** Preoperative serum fibrinogen and AKI Risk Following Cardiac Valve Replacement Surgery.

	95% CI	P Value
**Any AKI**		
Unadjusted OR	1.373 (1.236–1.525)	<0.001
Adjusted* OR	1.211 (1.100–1.333)	0.001
**Severe AKI**		
Unadjusted OR	1.439 (1.043–1.986)	<0.027
Adjusted* OR	1.193 (0.828–1.719)	0.343

*Note: Adjusting for age, sex, hypertension, diabetes mellitus, cerebrovascular disease, obesity, emergent surgery, surgery type, preoperative LVEF, eGFR, HB, and INR.

Abbreviations: acute kidney injury (AKI); odds ratio (OR); confidence intervals (CI).

### Risk prediction for postoperative AKI

**Figure 1:**
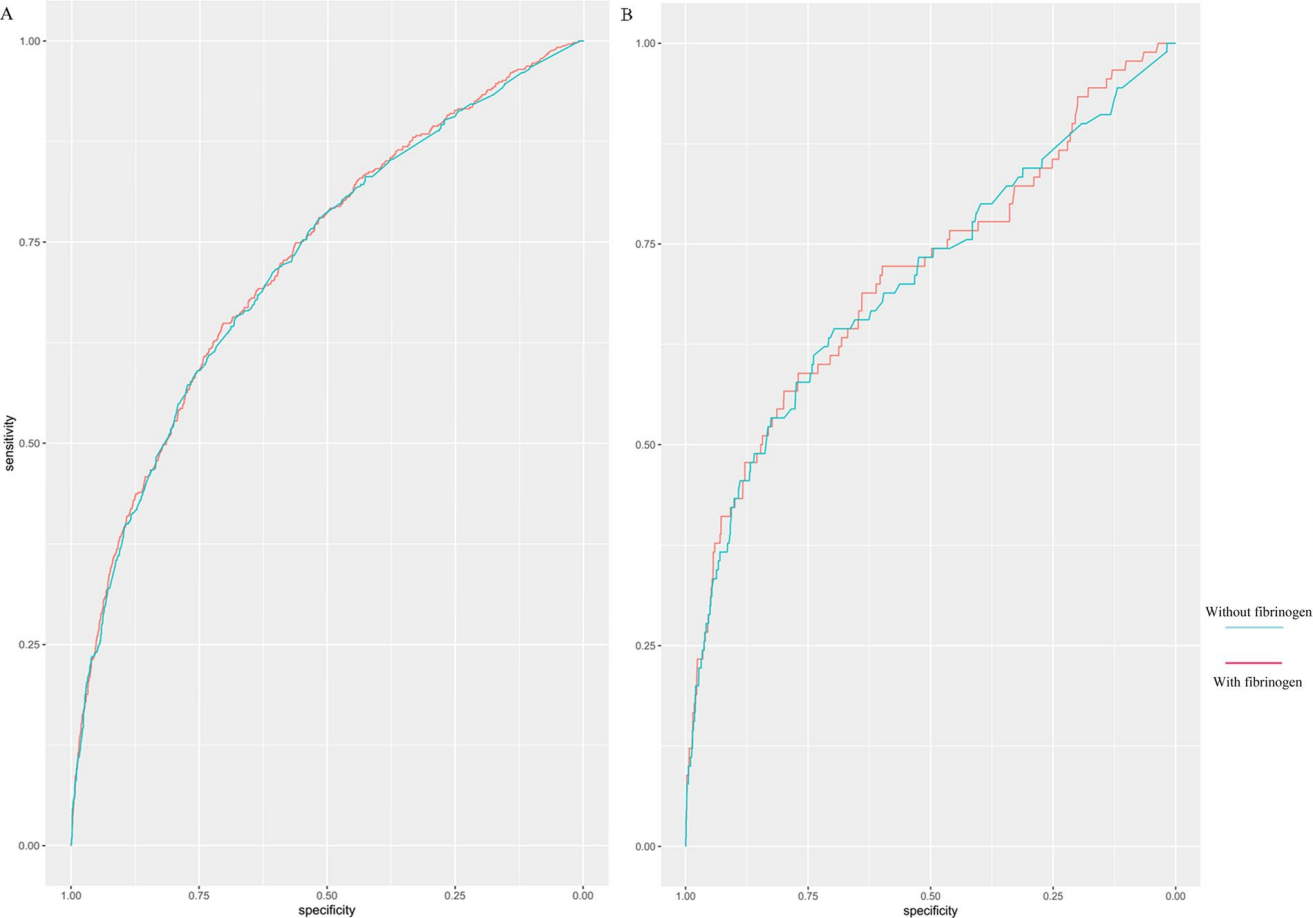
Incremental Changes to the Receiver Operator Characteristic (ROC) Curve. (**A**) Incremental changes to the ROC curve for AKI by adding serum fibrinogen to the multivariate risk analysis. (**B**) Incremental changes to the ROC curve for severe AKI by adding serum fibrinogen to the multivariate risk analysis.

The receiver operator characteristic (ROC) curve for the outcome of any AKI had a c-statistic of 0.72 using preoperative variables without serum fibrinogen. Addition of serum fibrinogen offered a marginally improved ROC (0.73, p < 0.001 for difference, Fig. 1A). For the severe AKI outcome, the c-statistic was 0.70 for preoperative variables without serum fibrinogen, and it increased to 0.71 with the addition of serum fibrinogen (p < 0.001 for difference, Fig. 1B).

The Net Reclassification Index (NRI) and the Integrated Discrimination Improvement (IDI) indices were used to assess whether serum fibrinogen improved AKI risk classification. The NRI was highly significant with a magnitude of 0.178 (95% CI, 0.084–0.271; p < 0.001), and the IDI was also highly significant (0.004; 95% CI, 0.001–0.007; p = 0.010). Among the 444 patients initially categorized as Intermediate risk without serum fibrinogen, 73 individuals were reclassified into lower (13%, n = 59) or higher (3%, n = 14) risk categories (Table 4).

**Table 4:** Comparison of Risk Classification for AKI.

Any AKI^a^	Risk Classification with fibrinogen*			
Risk Classification without fibrinogen	<25% risk	25–50% risk	>50% risk	All
<25% risk	2896	43	0	2939
25–50% risk	59	371	14	444
>50% risk	0	8	68	76
All	2955	422	82	3459

Values shown are number (AKI risk percentage).

*Risk classification is based on the following predictors: age, sex, hypertension, diabetes mellitus, cerebrovascular disease, obesity, emergent surgery, surgery type, preoperative LVEF, eGFR, HB, and INR.

Abbreviations: acute kidney injury (AKI).

### Association of serum fibrinogen with postoperative AKI after Synthetic Minority Oversampling Technique (SMOTE) subsampling

**Figure 2:**
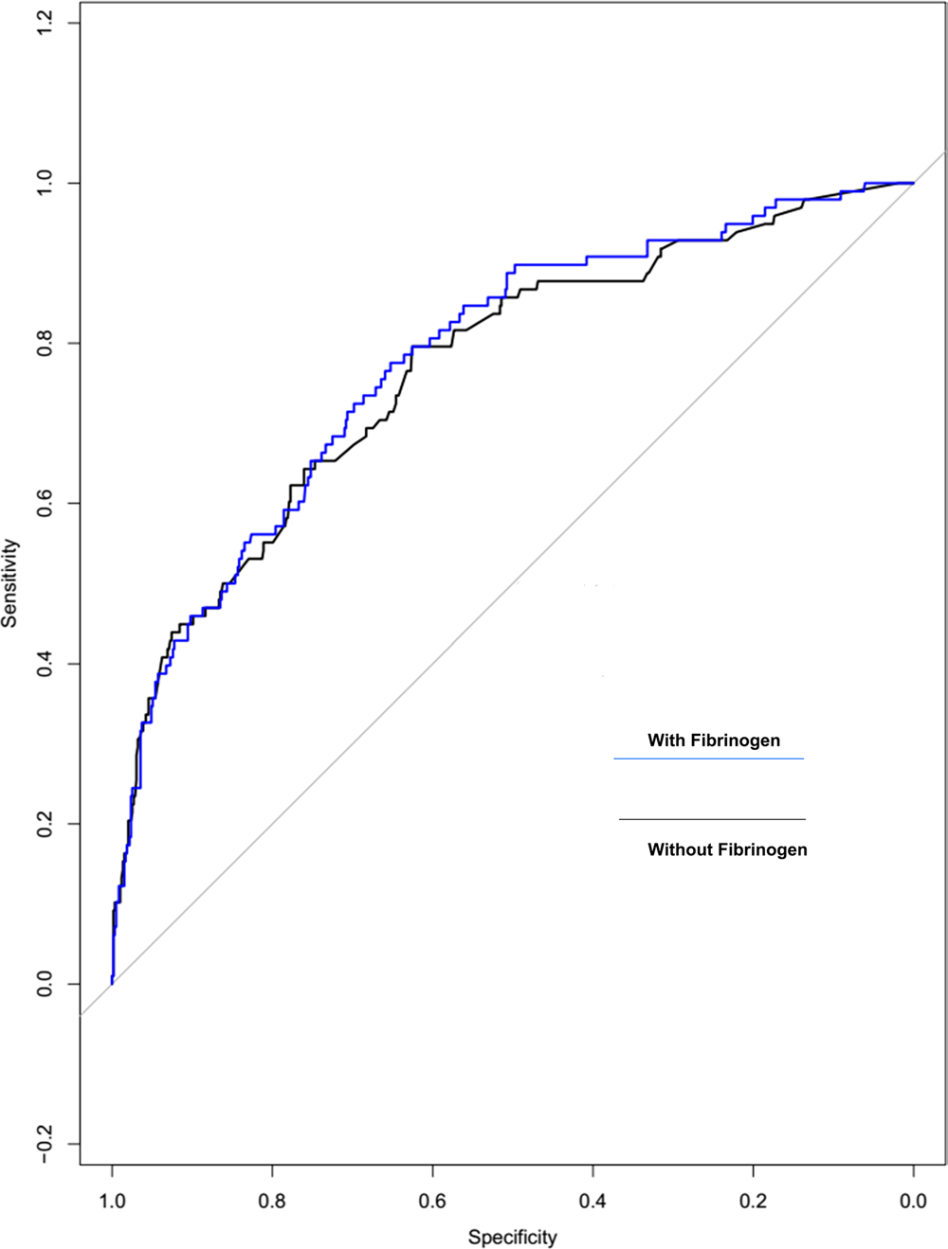
Incremental Changes to the Receiver Operator Characteristic (ROC) Curve after SMOTE subsampling. Incremental changes to the ROC curve for AKI by adding serum fibrinogen to the multivariate risk analysis.

Serum fibrinogen was independently associated with AKI grade 1 or higher (OR = 1.212, 95% CI 1.1089 to 1.347, p = 0.003) after SMOTE subsampling (Table 2). Meanwhile, the ROC curve for the outcome of AKI, after the addition of serum fibrinogen, had a c-statistic increasing from 0.77 to 0.78 (p < 0.001, Fig. 2).

## Discussion

Acute kidney injury is a common complication subsequent to cardiac surgery, and it causes long in-hospital stay and great long-term mortality risk. However, there continues to be a lack of therapeutic regimens to prevent or treat AKI. The present study demonstrates that preoperative serum fibrinogen concentration is associated with increased risk for postoperative AKI. For prediction of AKI, serum fibrinogen improved risk stratification with a slight increase in the c-statistic and a similarly slight improvements of the patient risk classification as NRI and IDI. Meanwhile, most preoperative risk factors, including sex, hypertension, diabetes mellitus, prior cardiac surgery, preoperative eGFR, and hemoglobin (HB) were independently associated with AKI, which was consistent with the previous studies^5–8^. There were no independent correlations with age, surgery type, cerebrovascular disease, obesity, emergent surgery, perioperative LVEF or the international normalized ratio (INR), which may due to the few older than 75 years old patients, emergent surgery and combined surgery type, and low incidences of obesity, cerebrovascular disease and poor cardiac function. Therefore, serum fibrinogen may have a promising role for preoperative stratification of patients with cardiac valve replacement surgery.

In our study, we observed an improvement in risk discrimination for the AKI outcome; a 1% increase in the c-statistic and a 17.8% increase in the NRI were both mild difference for a single biomarker. The risk scores existing could help surgeons and patients balance the risks and benefits of cardiac surgery. Owing to the severity of cardiac surgery outcomes, even mild improvements may contribute to risk adjustment for adverse outcomes, which is also clinical important. Therefore, if serum fibrinogen can help evaluate the risk of AKI or other adverse outcomes after cardiac surgery, then it might be valuable to patients and clinicians.

In cardiac surgery, perioperative bleeding remains a severe complication following cardiac surgeries that could lead to higher care costs and mortality risk^18^. Patients with a high bleeding risk due to CPB-related hemostatic defect, may necessitate multiple erythrocyte transfusions and re-exploration, both of which are associated with AKI^19^. Fibrinogen concentrate and its related products, such as fresh frozen plasma (FFP) and cryoprecipitate, are conventionally used as the standard replacement to control postoperative bleeding^12^. Several studies have found that the prophylactic fibrinogen concentrate infusion reduces the bleeding in cardiac surgery^13^. Jahangirifard *et al*. found that prophylactic transfusion of fibrinogen decreased postoperative bleeding and in-hospital stay in patients undergoing heart transplant, but there was no significant difference in length of ICU stay or mortality between the fibrinogen group and control group. Patients in the fibrinogen group had more AKI after surgery, which may explain its controversial clinical application^14^. In this study, we found that preoperative serum fibrinogen levels were significantly associated with AKI risk after cardiac valve replacement surgery. It may infer that either exogenous fibrinogen concentrate infusion, or endogenous baseline serum fibrinogen, may have association with postoperative AKI. Therefore, patients at risk of AKI after cardiac surgery, especially those with high baseline preoperative serum fibrinogen levels, should be cautious about using fibrinogen concentrate or its related products, such as FFP and cryoprecipitate during the perioperative period, or at least be critically evaluated prior to use.

Numerous studies have found that serum fibrinogen levels are associated with cardiovascular risk factors or cardiovascular events^20,21^. In addition, fibrinogen has been associated with a variety of crucial renal functions. For instance, fibrinogen was found as a sensitive and early diagnostic translational biomarker of AKI detection in patients with abdominal aortic aneurysm repair, due to its immunoreactivity in the kidney and urinary excretion^22^. Meanwhile, Celik *et al*. observed that an elevated procedural serum fibrinogen level was associated with the development of contrast-induced AKI^23^.

In terms of the pathophysiology of cardiac surgery-associated AKI, one main factor for the development of AKI is inflammation^4^. Fibrinogen is also recognized as an acute-phase response protein, and it increases several-fold during inflammation or tissue damage^15^. Previous study has found that fibrinogen stimulates chemotactic cell migration on binding to the leukocyte integrin receptor^24^. In addition, fibrinogen could stimulate mononuclear cells’ expression of proinflammatory cytokines, which suggesting that fibrinogen might be a marker of inflammatory factor^9^.

Regardless of the stimulating source or severity of the injury, almost all forms of tissue injury are associated with local activation of the coagulation system. Fibrinogen plays a key role in the acute phase response to tissue injury, where thrombin cleavage of fibrinogen integrates with the acute inflammatory response to inhibit tissue damage^15^. Therefore, preoperative serum fibrinogen may not simply reflect coagulation, but it may be a marker of multi-tissue damage. Elevated fibrinogen level is associated with increased blood viscosity, which may increase shear stress and damage endothelial function^25,26^. The increase in blood viscosity induced by fibrinogen will lead to renal tubular hypoperfusion and hypoxia, which cause AKI^27^. As an epiphenomenon, AKI may be a signal of adverse consequences from other affected organ systems as the result of multiple factors.

With regard to the underlying pathophysiological mechanisms, it has to be recalled that most of the patients undergoing cardiac surgery had been suffering from infections. Fibrinogen is playing an essential role not only in the systemic blood coagulation cascade, but in the acute phase response towards pro-inflammatory stimuli. Prophylactic fibrinogen increased the incidence of AKI, and the present study found that serum fibrinogen levels were associated with AKI. Therefore, whether perioperative infusion of fibrinogen concentrate could cause AKI after cardiac valve replacement surgery, especially in patients with high baseline preoperative serum fibrinogen levels, need further validation of randomized trials. The present study does have several limitations. First, retrospective analyses limited data quality. Unmeasured confounders are an important element in the retrospective study. Second, we did not discuss the intra- and postoperative events, which may affect the incidence of AKI. Our study was to evaluate patients at risk of AKI preoperatively so that effective prevention strategies may apply before or at the commencement of surgery. Third, since urine output data were not recorded, it was not used as the AKI definition in the present study. Fourth, the present cohort was recruited from a single center and single ethnicity, with a relatively young age, and only included the type of cardiac valve replacement surgery, which limited the applicability of our findings to other settings. Fifth, although there was an increase in the c-statistic for severe AKI, the use of IDI to reclassify categories of AKI risk was not significant. These results may due to the low morbidity of severe AKI (2.60%, n = 90). In addition, in our cohort, there was more than 50% women. However, the proportion of male patients in the postoperative AKI cohort was significantly higher than that in the non-AKI group, which was consistent with previous studies^8,19^.

In conclusion, in the present study, we found that preoperative serum fibrinogen levels were significantly associated with AKI risk. For the outcome of AKI, serum fibrinogen improved risk stratification based on changes in the c-statistic of the ROC curve and the reclassification of the categories of AKI risk. Our data imply that patients at risk of AKI after cardiac surgery, especially those with high baseline preoperative serum fibrinogen levels, should be cautious about using fibrinogen concentrate during the perioperative period, or at least be critically evaluated prior to use.

## Methods

### Study population

We retrospectively studied 3459 patients who underwent cardiac valve replacement surgery between January 2011 and September 2015 at the First Affiliated Hospital, College of Medicine, Zhejiang University. All patients underwent cardiopulmonary bypass. Patients, who underwent heart transplant and procedures for automated implantable cardioverter-defibrillator or left ventricular assist devices; those who required preoperative dialysis, preoperative extracorporeal membrane oxygenation, preoperative tracheostomy, or mechanical ventilation; those received the blood products preoperatively; those with liver disease and those with missing data, were excluded. The data collection and all experimental protocols in this study were approved by the Research Ethics Committee of the First Affiliated Hospital, College of Medicine, Zhejiang University approved the study (No. 20191059). All of the methods were carried out in accordance with the approved guidelines and relevant regulations. Written informed consent was obtained from all participants.

### Predictors

The primary evaluated parameter was the preoperative serum fibrinogen level, which were measured within 48 hours of surgery by the Clauss method using the BCS analyzer (Multifibren U; Siemens Healthcare, Erlangen, Germany). The normal range of serum fibrinogen level was 1.80–3.50 g/L. Co-variates for multivariable adjustment were obtained from the clinical records as well as the linked clinical inspection database of the hospital.

Covariate were chosen based on the previously published Cleveland Clinic^5^, the Metha^6^ and the Birnie scores^8^ for predicting AKI following cardiac surgery, as well as others related to surgery. The covariates were: age, sex, co-morbidities (hypertension, diabetes mellitus, cerebrovascular disease, obesity), surgery characteristics (elective or emergency; valve replacement surgery only, combined valve replacement surgery and others; prior cardiac operation), and preoperative markers (LVEF; eGFR; serum creatinine; HB; INR). Age was grouped into three categories: ≤60 years, 61 to 75 years, and >75 years. Obesity was defined as body mass index (BMI) ≥28.00 kg/m^2^. Preoperative LVEF was grouped as good ≥50.00%, fair 30.00 to 49.99%, or poor <30%. eGFR was grouped as ≤30.00, 30.01 to 60.00, 60.01 to 90.00, and >90 mL/min/1.73m^2^. HB was grouped as ≤100, 101 to 120, and >120 g/L. INR was grouped as ≤0.90, 0.91 to 1.10, and >1.10.

### Outcomes following surgery

The primary outcome was AKI, defined as AKI stage-1 or higher based on the Kidney Disease: Improving Global Outcomes (KDIGO) Guidelines^28^. AKI stage-1 was defined as an increase from a baseline of ≥26 μmol/L of postoperative creatinine or an increase of 1.5 to 1.9 times the preoperative creatinine within 7 days; stage-2 was an increase of 2.0 to 2.9 times the preoperative creatinine; stage-3 AKI was an increase ≥3 times the preoperative creatinine or an increase to ≥354 μmol/L or when the patient commenced renal replacement therapy (RRT). RRT was administered for uraemia, volume overload, or biochemical abnormalities, according to institutional protocols. We evaluated a secondary outcome of severe AKI (AKI stage-2 or higher).

### Statistical analysis

We univariably compared patients with and without AKI on the risk factors using unpaired t-tests for continuous variables and χ2 tests or Fisher’s exact tests for categorical variables. Multivariate logistic regression analyses were used to determine the associations of fibrinogen with AKI. The models were adjusted for demographics, co-morbidities, procedure variables and the preoperative marker as listed above.

To evaluate the added effect of serum fibrinogen on risk discrimination, we used ROC curve and calculated the c-statistic. The c-statistic was determined for the multivariate model without serum fibrinogen, and repeated with serum fibrinogen. This process was repeated for the outcome of severe AKI.

As a second step to assess the impact of serum fibrinogen on AKI risk prediction, we determined the NRI and IDI indices^29,30^. The NRI evaluated the appropriateness of reclassification between models before and after serum fibrinogen was added, tabulating the frequency of appropriate versus inappropriate reclassification; a significant P value indicated that significantly more patients were being reclassified appropriately than inappropriately. By contrast, the IDI determined how much an individual’s predicted risk changed with the use of different models^31^.

For NRI analyses, we initially categorized all patients as being at a low (<25%), medium (25–50%), or high (>50%) AKI risk based on the prediction model that incorporated all of the clinical variables listed above. The AKI risk was re-calculated with adding serum fibrinogen, and patients were re-classified into low-, medium- and high-risk groups. Then the NRI was determined to describe the AKI risk for individuals who were moved to lower and higher groups by serum fibrinogen.

Data in the non-AKI case outnumbered those in the AKI case by a ratio of approximately 7:1. After data cleaning and feature processing, the SMOTE was applied to balance the AKI and non-AKI classes^32^. The SMOTE created new minority data by interpolation within the available minority data via bootstrap sampling and data generation via the κ-nearest neighbors algorithm. The K parameter, which determines the numbers of closest neighbors considered with each SMOTE iteration, was set to 5. Random allocation was used to assign the data to the training and testing sets at a ratio of 4:1. All analyses were performed in R programming language version 3.5.3.

## Data Availability

The datasets used and analyzed during the current study are available from the corresponding author on reasonable request.
